# Macrophage Migration Inhibitory Factor Predicts Outcome in Complex Aortic Surgery

**DOI:** 10.3390/ijms18112374

**Published:** 2017-11-09

**Authors:** Alexander Gombert, Christian Stoppe, Ann Christina Foldenauer, Tobias Schuerholz, Lukas Martin, Johannes Kalder, Gereon Schälte, Gernot Marx, Michael Jacobs, Jochen Grommes

**Affiliations:** 1 European Vascular Center Aachen-Maastricht, Department of Vascular Surgery University Hospital Aachen, RWTH Aachen University, Pauwelsstraße 30, 52074 Aachen, Germany; jkalder@ukaachen.de (J.K.); mjacobs@ukaachen.de (M.J.); jgrommes@ukaachen.de (J.G.); 2 Department of Intensive Care and Intermediate Care, University Hospital Aachen, RWTH Aachen University, 52074 Aachen, Germany; cstoppe@ukaachen.de (C.S.); lmartin@ukaachen.de (L.M.); gmarx@ukaachen.de (G.M.); 3 Department of Medical Statistics, University Hospital Aachen, RWTH Aachen University, 52074 Aachen, Germany; afoldenauer@ukaachen.de; 4 Department of Anaesthesia and Intensive Care, University of Rostock, 18059 Rostock, Germany; tobias.Schuerholz@med.uni-rostock.de; 5 Department of Anesthesiology, University Hospital Aachen, RWTH Aachen University, 52074 Aachen, Germany; gschaelte@ukaachen.de

**Keywords:** macrophage migration inhibitory factor, thoracoabdominal aortic aneurysm, intensive care unit, survival

## Abstract

The perioperative inflammatory response is associated with outcome after complex aortic repair. Macrophage migration inhibitory factor (MIF) shows protective effects in ischemia-reperfusion (IR), but also adverse pro-inflammatory effects in acute inflammation, potentially leading to adverse outcome, which should be investigated in this trial. This prospective study enrolled 52 patients, of whom 29 (55.7%) underwent open repair (OR) and 23 (44.3%) underwent endovascular repair (ER) between 2014 and 2015. MIF serum levels were measured until 72 h post-operatively. We used linear mixed models and ROC analysis to analyze the MIF time-course and its diagnostic ability. Compared to ER, OR induced higher MIF release perioperatively; at 12 h after ICU admission, MIF levels were similar between groups. MIF course was significantly influenced by baseline MIF level (*P* = 0.0016) and acute physiology and chronic health evaluation (APACHE) II score (*P* = 0.0005). MIF level at 24 h after ICU admission showed good diagnostic value regarding patient survival [sensitivity, 80.0% (28.4–99.5%); specificity, 51.2% (35.1–67.1%); AUC, 0.688 (0.534–0.816)] and discharge modality [sensitivity, 87.5% (47.3–99.7%); specificity, 73.7% (56.9–86.6%), AUC, 0.789 (0.644–0.896)]. Increased perioperative MIF-levels are related to an increased risk of adverse outcome in complex aortic surgery and may represent a biomarker for risk stratification in complex aortic surgery.

## 1. Introduction

Macrophage migration inhibitory factor (MIF) is a proinflammatory chemokine-like cytokine that plays critical roles in multiple inflammatory conditions [1,2]. Cytokines are multifunctional mediators that modulate immune activity via receptor-mediated pathways. MIF exerts predominantly pro-inflammatory effects in acute and chronic inflammation, as found in acute kidney or lung injury, sepsis, colitis, or atherosclerosis [3,4,5,6]. For example, in atherosclerosis, MIF activates CXCR2 and CXCR4, playing a major role in regulating inflammatory cell recruitment. In contrast to these negative pro-inflammatory and pro-atherogenetic effects, MIF release during myocardial ischemia/reperfusion or cardiac surgery is reportedly associated with reduced organ failure and improved patient outcome [7,8,9].

Both open and endovascular thoracoabdominal aortic aneurysm (TAAA) repair is related to high rates of morbidity and mortality [10,11,12]. Open repair (OR) requires aortic clamping, which may be associated with risk of spinal cord ischemia (SCI), acute kidney injury (AKI), mesenteric ischemia, or lung injury [13,14]. Although outcome can be improved by distal perfusion with left heart bypass, adapted aortic clamping, and selective organ perfusion [15,16,17], this procedure remains associated with substantial mortality and morbidity. Compared to open techniques, new endovascular methods reduce surgical trauma by avoiding thoracoabdominal access, potentially enabling treatment of patients ineligible for open surgery. However, endovascular repair (ER) of complex TAAA still carries mortality rates of 2–21% [18,19], and is associated with morbidity (e.g., AKI or SCI) [13].

There exists a need for new biomarkers to potentially improve the understanding of the acute inflammation during complex aortic aneurysm repair and to help vascular surgeons to predict postoperative complications and identify high-risk patients. Few biomarkers have been assessed in fields of open or endovascular TAAA repair in terms of inflammation reaction and post-interventional adverse outcome. MIF has been described as a promising outcome parameter, which is related to negative pro-inflammatory effects in current clinical studies [4,5]. As a potential biomarker for this purpose, in our present study, we investigated the intriguing role of MIF during complex aortic surgery.

## 2. Results

### 2.1. Study Population

This study included 52 patients who underwent open surgery (29 patients; 55.7%) or endovascular surgery (23 patients; 44.3%) for treatment of a TAAA. Of the treated TAAAs, 40.3% (*n* = 21) were type II, 4% (*n* = 2) were type III, and 55.7% (*n* = 29) were type IV. A total of 12 patients (23.2%) were women. No patient included in this study suffered from connective tissue disease. The mean age was 64.5 ± 10.4 years (range, 43–85 years), and patients receiving endovascular repair were significantly older (*P* < 0.0001). Table 1 presents detailed patient information.

### 2.2. Complications, Re-Interventions, and Mortality

Baseline serum creatinine levels were significant higher in the endovascular group (1.14) compared to the open surgery group (0.92; *P* = 0.0389). However, the endovascular subgroup did not show a higher rate of AKI (open, 27.6% vs. endovascular, 26.1%; *P* = 0.2449) or AKI requiring dialysis (open, 17.2% vs. endovascular, 26.1%; *P =* 0.506). A total of 26.9% of the patients (*n* = 14) developed AKI, of whom, 21.2% (*n* = 11) required temporary dialysis. Among these patients, 8 completely recovered, while 3 (5.7%) required permanent dialysis.

No cases of acute myocardial infarction were reported. Ten patients (19.2%) developed pneumonia.

Six patients (11.5%) developed sepsis, of which five cases were related to pneumonia, and one case was related to small intestine ischemia following embolization during open type III repair. A total of 12 patients (23%) required re-intubation, of whom 10 (19.2%) underwent tracheotomy.

A total of 12 patients (23%) required re-intubation, of whom 10 (19.2%) underwent tracheotomy.

Six patients (11.5%) required surgical revisions. Three of these cases underwent access-related wound revision. Two patients needed surgical re-intervention due to hemothorax. One case of small intestine ischemia required multiple revisions, including bowel resection. Details are presented in Table 1.

Within 90 days after TAAA surgery, 9.6% of the patients (*n* = 5) died. Two patients died due to pneumonic sepsis, and one due to cerebral bleeding. One patient developed small intestine ischemia associated with pancreas necrosis, and peritonitis appeared during the hospital stay. The fifth patient had a type II TAAA, which was successfully treated by type IV repair as a first step, but then suffered thoracic aortic rupture during the recovery phase before completion of his second surgical therapy.

All patients who survived the first 90 days after surgery were contacted between December 2015 and January 2016. Mean follow-up was 13.2 months (±5.3; range, 2–20 months).

### 2.3. Higher MIF Release after OR vs. ER

**Figure 1:**
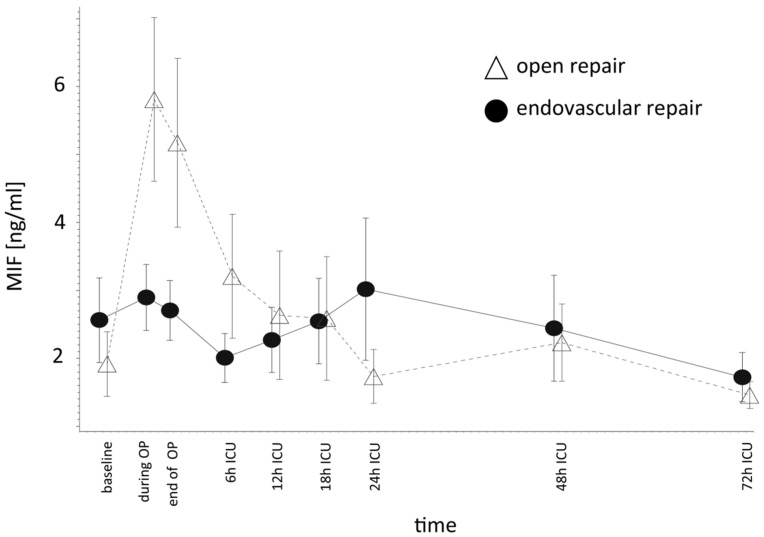
Macrophage migration inhibitory factor (MIF) release during aortic surgery. Compared to endovascular repair, the open procedure induced higher perioperative MIF release, and this difference persisted up to the first hours after intensive care unit (ICU) admission. Endovascular repair induced only a moderate perioperative MIF increase; MIF values are presented as median with interquartile range (ng/mL).

Compared to ER, OR induced higher perioperative MIF levels. MIF levels remained higher in the OR group until 6 h after ICU admission. The OR showed the highest MIF levels at 1 h after aortic clamping. ER induced a more modest increase of MIF. At 12 h after ICU admission, MIF levels decreased to within the range of preoperative values in both groups (Figure 1). The mean logarithmized MIF levels also tended to be higher in the OR group compared to the ER group, but this value was only significantly higher in the OR group at the time of ICU admission (*P =* 0.0242) (Tables S1 and S2).

### 2.4. Survival Rate and Discharge Modality

**Figure 2:**
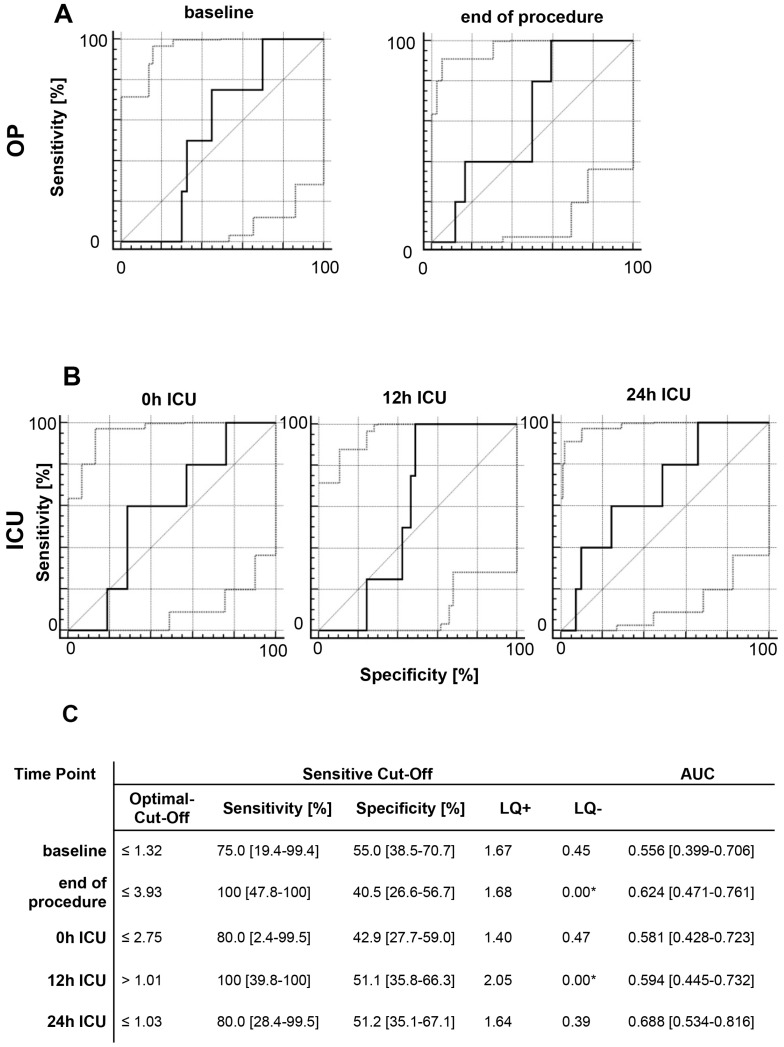
ROC analysis of perioperative MIF levels and patient survival. ROC analysis was performed to evaluate the diagnostic capacities of (**A**) perioperative MIF levels and (**B**) MIF levels during ICU admission with regard to survival. If an elevated MIF value indicates that the patient is likely to die after surgery, the ROC curve should be farther from the bisecting line (Sensitivity = 1 − Specificity). (**C**) Sensitivity (Se), specificity (Sp), likelihood ratios (LR +/−), and area under the curve (AUC) are reported for either the Youden optimal cut-off (maximize Se + Sp − 1) or for a sensitivity cut-off of at least 75%. * Good-to-moderate diagnostic quality: LQ+ of >3 and LQ− of <0.3. * Excellent diagnostic quality: LQ+ of >10 and LQ− of >0.1.

**Figure 3:**
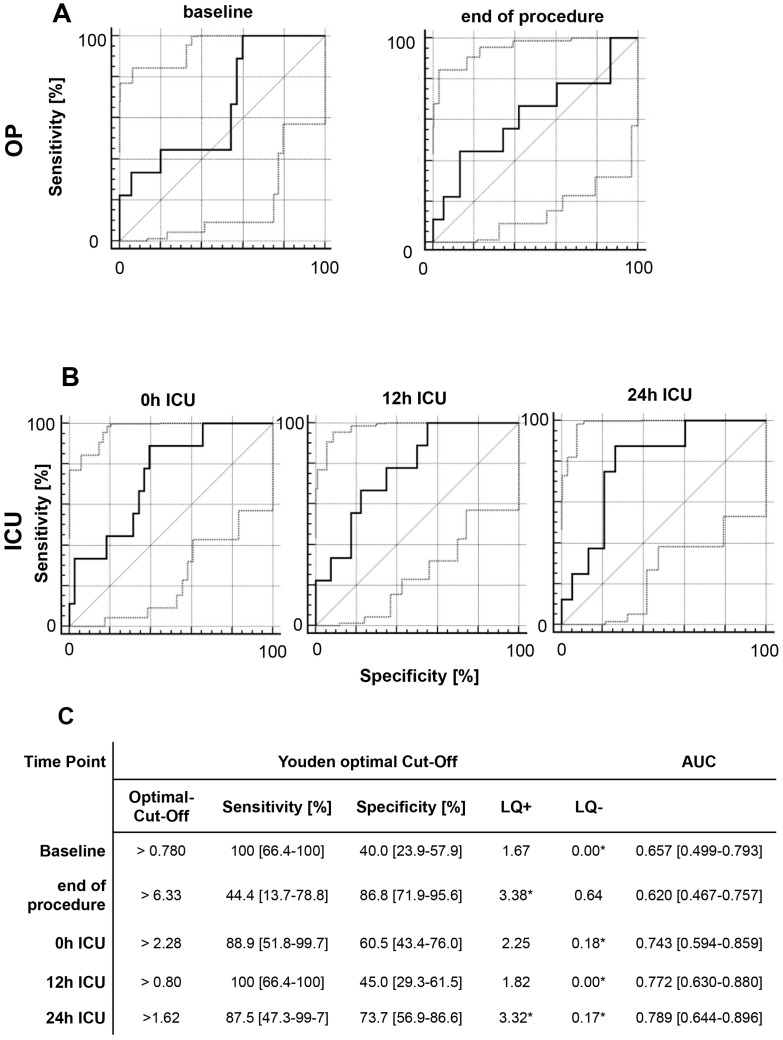
ROC analysis of perioperative MIF levels and patient discharge modality. ROC analysis was performed to evaluate the diagnostic capacities of (**A**) perioperative MIF levels and (**B**) MIF levels during ICU admission with regard to discharge modality. If an elevated MIF value indicates an adverse discharge modality, the ROC curve should be farther from the bisecting line (Sensitivity = 1 − Specificity). (**C**) Sensitivity (Se), specificity (Sp), likelihood ratios (LR +/−), and area under the curve (AUC) are reported for either the Youden optimal cut-off (maximize Se + Sp − 1) or for a sensitivity cut-off of at least 75%. * Good-to-moderate diagnostic quality: LQ+ of >3; LQ− of <0.3. * Excellent diagnostic quality: LQ+ of >10 and LQ− of >0.1.

Among the patients in our study, 9.6% (*n* = 5) died within 90 days after TAAA surgery. The OR and ER subgroups did not differ with regard to survival or discharge modality. We investigated if MIF values could predict the survival rate and discharge modality by analyzing the MIF values of survivors and non-survivors at early time points after treatment. In our analysis of data from five time-points, we detected a good-to-moderate association between elevated MIF levels and a reduced survival rate. At 12 h after ICU admission, we found a sensitivity of 100% (39.8–100%), specificity of 51.1% (35.8–66.3%), and area under curve (AUC) of 0.594 (0.445–0.732), and at 24 h after ICU admission, sensitivity was 80.0% (28.4–99.5%), specificity was 51.2% (35.1–67.1%), and AUC was 0.688 (0.534–0.816) (Figure 2). Regarding discharge modality, we defined discharge via a normal ward as favorable modality, and discharge via the ICU or weaning ward or death as adverse modality. At every time-point, elevated MIF levels showed good-to-moderate diagnostic capability for an adverse discharge modality. MIF level at 24 h after ICU admission showed particularly good diagnostic quality, with a sensitivity of 87.5% (47.3–99.7%), specificity of 73.7% (56.9–86.6%), and AUC of 0.789 (0.644–0.896) (Figure 3).

### 2.5. Acute Kidney Injury

**Figure 4:**
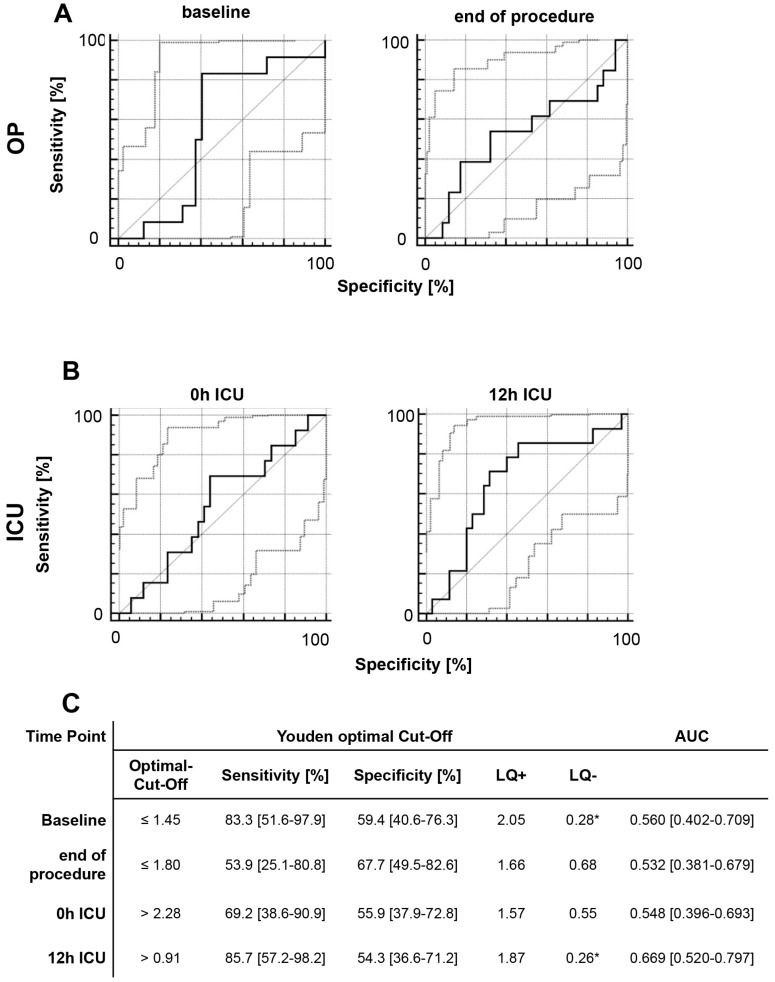
ROC analysis of perioperative MIF levels and acute kidney injury. ROC analysis was performed to evaluate the diagnostic capacities of (**A**) perioperative MIF levels and (**B**) MIF levels during ICU admission with regard to acute kidney injury (AKI) based on a serum creatinine increase of >50% within 48 h after ICU admission. If an elevated MIF value indicates AKI, the ROC curve should be farther from the bisecting line (Sensitivity = 1 − Specificity). (**C**) Sensitivity (Se), specificity (Sp), likelihood ratios (LR +/−), and area under the curve (AUC) are reported for either the Youden optimal cut-off (maximize Se + Sp − 1) or for a sensitivity cut-off of at least 75%. * Good-to-moderate diagnostic quality: LQ+ of >3 and LQ− of <0.3. * Excellent diagnostic quality: LQ+ of >10 and LQ− of >0.1.

We identified a moderate correlation between baseline MIF level and post-interventional AKI, with a sensitivity of 83.3% (51.6–97.9%), specificity of 59.4% (40.6–76.3%), and AUC of 0.560 (0.402–0.709) (Figure 4). We analyzed the predictive power of MIF separately in the OR and ER subgroups, and found that elevated MIF level at 24 h after ICU admission was significantly correlated with AKI after OR (*P =* 0.0476). MIF level was not significantly correlated with AKI at any other time-point in either subgroup (Table S3).

### 2.6. Multivariate Analysis of MIF Release Following OR and ER

To assess the potential influence of baseline characteristics and surgery-related data on perioperative MIF release, we assessed different continuous and categorical variables with regard to their impact on MIF release after TAAA surgery. Pre-operative time-points were excluded from analyses scrutinizing the influence of the applied treatment modality on MIF levels. Using a linear mixed model, with a univariate approach to evaluate various factors, we identified the logarithmized baseline MIF level as a factor influencing the MIF course for all patients (*P* = 0.0016). With regard to continuous variables, APACHE II score was significantly correlated with MIF levels (*P* = 0.0005). Inclusion of age, BMI, and gender did not improve the power of the model (Table 2).

We subsequently evaluated MIF measurement time-points, treatment modalities, and baseline MIF levels in a multivariate analysis using a reduced set of effect parameters from the univariate analysis (Table 3). The multivariate model revealed that baseline MIF level had a significant impact on the further course of MIF (*P* = 0.0010). With regard to surgery method, OR showed a trend of association with higher MIF levels, but this association was not significant (*P* = 0.0677). Analysis of the time effect showed that log (MIF) levels significantly differed over time (*P* < 0.0001). More importantly, OR induced higher MIF levels in comparison to ER (*P* = 0.0121), and the mean log (MIF) levels at ICU admission significantly differed between the OR and ER subgroups (*P* = 0.0242) (Table S4). 

Evaluation of baseline characteristics revealed that patients suffering from coronary artery disease (CAD) showed significant lower MIF levels after ICU admission in univariate analysis (*P* = 0.0395); however, this effect could not be confirmed in the multivariable model (*P* = 0.2790). Comparison of MIF levels between patients with and without CAD for different time-points revealed a significant CAD by time interaction effect (*P* = 0.0395), but the log (MIF) levels did not differ between patients with and without CAD at any time-point. In the comparison of MIF levels between CAD and non-CAD patients, the baseline MIF level and the overall time effect remained significant (*P* = 0.0021 and 0.0001, respectively).

## 3. Discussion

To our knowledge, the present study is the first to reveal an association between elevated MIF levels and adverse events or mortality after complex aortic surgery. MIF is a proinflammatory chemokine-like cytokine that plays critical roles in several inflammatory conditions [1,2]. For example, MIF promotes atherosclerosis by activating proinflammatory atherogenic pathways [7]. Accordingly, elevated MIF levels have been previously described in patients with CAD [7,20], and we presently report similar results independent of the treatment modality. As CAD is associated with a state of chronic inflammation, it is likely that a stimulus for MIF release has a greater effect in patients with CAD than in those without CAD. Notably, Gong et al. also reported overexpression of MIF in patients suffering from CAD [21].

Previous studies have also evaluated MIF in patients undergoing cardiac surgery. Stoppe et al. reported that high intraoperative MIF levels were associated with a reduced extent of organ injury after cardiac surgery [22]; however, these findings were not confirmed by our present data. In contrast, we observed that high MIF levels at 24 h after surgery were associated with a higher incidence of AKI. This discrepancy could potentially be due to the different time-points analyzed in these studies. Stoppe et al., who focused on perioperatively measured MIF-levels during their study, demonstrated beneficial effects of intraoperative MIF release, which is in line with recent experiment findings [22]. On the other hand, we revealed that postoperative MIF levels were associated with adverse effects. This could be a result of MIF’s pro-migratory effect on diverse immune cells, which may further amplify the overall inflammatory response and has been demonstrated as well in previous clinical studies [23,24]. Moreover, the negative effects of MIF revealed in our study could be related to a prolonged inflammatory response that is associated with adverse outcome, whereas a short-term MIF release induced by ischemia/reperfusion can reduce organ failure as shown after cardiac surgery [22].

Stefaniak reported that MIF elevation on the first day after liver transplantation predicts acute kidney injury [4]. The surgical trauma from cardiac surgery, liver transplantation, or aortic surgery induces a sepsis-like immune response, which can lead to severe systemic inflammation and organ failure. In our study, we addressed perioperative MIF release and its clinical significance in patients undergoing open and endovascular aortic repair surgery. Open aortic surgery is associated with surgical trauma comparable to cardiac bypass surgery with the use of extracorporeal circulation and aortic cross-clamping. Previous studies have investigated the inflammation during and following extended aortic surgery. Fiane et al. reported complement activation after open TAAA repair without extracorporeal circulation, but not after infrarenal OR or thoracic ER [25]. Welborn et al. measured the release of TNF-alpha, IL-6, IL-8, IL-10, and shed TNF receptors during TAAA repair, and revealed that increased levels of TNF-α and IL-6 were associated with more frequent development of multiple organ dysfunction [26]. However, in these studies, TAAA surgery was performed without use of extracorporeal circulation.

Compared to ER, OR was associated with higher MIF release during and at the end of the operation. In OR, MIF level peaked after aortic clamp removal and at the end of aortic repair. On the other hand, in ER, the highest MIF level was observed after the intervention, and this peak was much smaller than the MIF peak in OR. These findings indicated a different mechanism of MIF release during ER vs. OR. In line with our results, Moris et al. described a higher incidence of systemic inflammation in open surgical treatment of abdominal aortic aneurysm than in endovascular repair [27,28]. Although the potential mechanisms for delayed MIF increase cannot be addressed in a clinical study, we speculate that the delayed MIF increase after ER may result from MIF being released from mainly pro-inflammatory immune cells. In contrast, the intraoperative MIF release in OR patients is directly triggered by ischemia/reperfusion, resulting in the well-documented prompt release of MIF from endothelial cell [29,30]. Although the ER procedure avoids major surgical trauma, up to 30% of patients develop non-infectious inflammation with fever and leukocytosis [31]. This so-called post-implantation syndrome is associated with an increased incidence of cardiovascular events [32], and may be a reason for the delayed MIF release after ER. Additionally, prolonged MIF release could be a predictor of prolonged inflammation after both OR and ER. As the ER group in this study was significantly older, a potential influence of the patients’ age on the different pattern of release could be discussed.

The perioperative MIF levels were influenced by extreme MIF levels, with a maximum concentration of 29.59 µg/mL, which increased the length of the confidence interval. These high levels may explain the absence of significant differences for all perioperative time-points, as well as the partially weak correlation between MIF levels and patient outcome.

In our study, we also assessed how different influencing factors impacted MIF release. Only the baseline MIF value and the APACHE II score showed significant impact on the measured MIF levels. Multivariate analysis confirmed that baseline MIF values were significantly correlated with MIF levels. In terms of the association of elevated MIF levels with adverse discharge and decreased survival rate, the clinical relevance of our present findings is underscored by the association of elevated MIF levels with the APACHE II score—a diagnostic tool for the probability of surveillance of critical ill patients.

With regard to patient outcome, we examined organ failure and survival rate. We found that elevated MIF levels at 12 and 24 h after ICU admission showed a good-to-moderate correlation with adverse outcome in terms of survival rate and, especially, discharge modality. These findings concur with the results of Pohl et al., which demonstrated that elevated MIF levels at 24 h after ICU admission were associated with mortality rate in critical ill patients [33]. Our present data also showed that MIF levels were weakly correlated with AKI. MIF release is determined by different factors, and our present study cohort is inhomogeneous, which may have contributed to this weak correlation. Furthermore, the weak correlation with patient outcome may also be explained by the high variation among different MIF levels.

Separate analysis of the open surgical and endovascular subgroups generated no significant results. However, prior studies have described strong correlations of AKI with MIF levels [4,34], which may indicate that MIF has overall strong functional relevance in the pathophysiology during endovascular and open TAAA repair. With regard to acute kidney injury, our present results did not confirm these findings. As presented by Pohl et al., renal replacement therapy can potentially eliminate elevated levels of cytokines, such as MIF, in patients suffering from septic shock, resulting in improved survival rates [35]. In terms of the elevated MIF-levels and their correlation with patients’ outcome, a clinical applicability of specific MIF-antibodies, to counteract the observed disease aggravating properties, might be a promising strategy to improve these patients’ outcomes.

## 4. Material and Methods

This study included patients undergoing surgery for TAAA. Exclusion criteria were age of <18 years, pregnancy, chronic kidney disease requiring dialysis, physical or mental disability, and emergency procedures. All patients gave preoperative written informed consent. The local ethics committee approved this study (University Hospital Aachen EK004/14). Written informed consent was obtained preoperatively from all subjects. This study was performed in accordance with the Declaration of Helsinki in its actual form. The study is registered at clinicaltrials.gov (NCT03093857). The datasets supporting the conclusions of this article are included within the article and its supplemental files.

### 4.1. Data Collection

Data were prospectively collected and continuously analyzed. Baseline characteristics were assessed and documented on the first day of enrollment. The Acute Physiology and Chronic Health Evaluation II (APACHE II) score were determined daily to assess organ dysfunction at admission on intensive care unit (ICU) as well as after 24 and 48 h after admission on ICU. Blood and urine samples were collected at 12 predefined time intervals: baseline; before, 30 min after, and 60 min after clamping or contrast-solution application; end of procedure; ICU admission; and at 6, 12, 18, 24, 48, and 72 h after ICU admission.

The samples were centrifuged for 10 min and stored at −80 °C afterwards until further processing by Enzyme Linked Immunosorbent Assay was performed according to the manufacturer’s instructions. The procedure has been described before [7].

We assessed AKI based on serum creatinine and urine output in accordance with the RIFLE criteria [36]. AKI was defined by reduced kidney function with an increase of serum creatinine to >26.4 µmol/L or to >50% higher than the lowest pre-intervention value (baseline), as recommended in current guidelines [37].

We analyzed the course of MIF following open and endovascular repair of TAAA. We additionally assessed how elevated MIF levels were associated with patient outcome, AKI, survival rate, and adverse discharge modality. Adverse discharge was defined as discharge from hospital via weaning or death. Additional methods with details regarding the open and endovascular procedures are available online in the Supplemental Data (Details of the Surgical Procedure S1).

### 4.2. Statistical Analyses

No power analysis was conducted before the beginning of this study. Till now, the clinical relevance of MIF and its changes after open and endovascular TAAA repair have not been described as far as we are concerned. The presented data should be primary considered as hypothesis generating.

Continuous variables are expressed as mean ± standard deviation (SD) or 95% confidence interval. Variables showing heavily skewed distributions are instead expressed as median with 0.25 quantile (Q1) and 0.75 quantile (Q3). Categorical variables are expressed as absolute frequency and percentage. Some measurements were missing completely at random, and no systematic bias was detected. All available data was included in the analysis. Reduced sample sizes are reported accordingly. Based on the sample size a separate analysis of each type of TAAA repair in the OR and ER group regarding the perioperative MIF-release was not conducted. MIF level showed a skewed distribution, so the exact Wilcoxon signed-rank test was used to compare MIF elevation between single time-points in relation to various factors (Table S3). For the comparison of the surgery methods, these were estimated from a linear mixed model with transformed data described below (Table S2). Since the event rates were often small, we used an exact Fisher’s test to make comparisons between frequencies.

We used linear models with repeated measures to evaluate how certain metabolic factors impacted MIF levels. Only measurements after clamping (OR)/contrast solution exposition (ER) were considered as outcome for the analysis; baseline measurement influence was evaluated as a covariable.

The response parameter MIF was logarithmized to meet the model requirements. We accounted for the small sample size using Kenward-Rogers adjustment, and assumed an unstructured covariance matrix. Model fit was evaluated using residual plots. For univariate analysis, we considered the fixed time effect (repeated factor) and a random intercept as a base model. Effects were modeled in univariate analysis by extending the base model; for these, we report only the results of the additionally modeled effects. For the multivariable analysis of MIF, we extended the base model by the logarithmized MIF levels at baseline, the surgery method effect, and the fixed effect of time by surgery method interaction. We did not include effects of serum creatinine and APACHE II since they correlated and, more importantly, were not recorded for several time-points. Using backward selection, we also excluded age, BMI, and gender because they did not improve the model fit according to Akaike’s information criterion (AIC). We then applied the same model switching the surgery method effect with the effect of CHD (coronary heart disease) and diabetes. We did not consider all three factors (surgery method, CHD, and diabetes) together in one model due to the small sample size.

For all fixed effect variables, we report the estimated slope, its standard deviation (SD), degrees of freedom (DF), the test statistic (*t* value), and the *P* value. Since we considered more than two time-points, we report the *P* value of the overall F-Test (type 3) for the overall time effect together with two degrees of freedom (Num DF/Den DF) and the F-statistic (*F* value). An effect in the statistical model was considered significant if the corresponding *P* value fell below the 5% margin. Since this was an explorative study, we did not perform alpha adjustment. ROC analysis was performed to evaluate the diagnostic capacity of MIF with regard to patient survival, direct discharge category (favorable/adverse), need for tracheotomy (at surgery), and AKI (serum creatinine increase of >50% within 48 h after ICU admission). Sensitivity (Se), specificity (Sp), likelihood ratios (LR +/−), and AUC (area under the curve) are reported for either the Youden optimal cut-off (maximize Se + Sp − 1) or for a sensitivity cut-off of at least 75%. ROC curves are plotted, with the 95% confidence interval indicated by dotted lines. For fixed cut-offs, diagnostic quality was evaluated using the likelihood ratio following the method of Sackett et al., with good-to-moderate diagnostic quality indicated by an LR+ of >3 and an LR− of <0.3, and excellent diagnostic quality by an LR+ of >10 and an LR− of >0.1 [38]. Statistical analysis was performed using SAS for Windows, Version 9.4 (SAS Institute, Cary, NC, USA). “Proc Mixed” was used for repeated measure analysis. ROC analysis was performed using MedCalc for Windows, version 12.7.7.0 (MedCalc Software, Ostend, Belgium).

## 5. Conclusions

Open and endovascular TAAA repair resulted in different time course of MIF release. Postoperatively elevated MIF levels were associated with worse patient outcome, indicating that MIF has disease-aggravating effects. MIF shows promise as a potential biomarker for adverse events in patients undergoing either open or endovascular TAAA repair, representing a potential future therapeutic target for specific anti-inflammatory strategies in these patients.

## Figures and Tables

**Table 1:** Patient Characteristics According to Surgery Method.

	All Patients	Open Surgery	Endovascular Surgery	*P* Value ^#^
	(*n* = 52)	(44.2%; *n* = 29)	(55.8%; *n* = 23)	
**Patients characteristics and treatment**				
**Age, years**	64.5 ± 10.4	59.8 ± 10.7	70.48 ± 6.17	0.0001 *
**Male gender**	39 (75.0%)	22 (75.9%)	17 (73.9%)	0.8719
**BMI**	27.1 ± 3.9	26.4 ± 4.0	28.0 ± 3.8	0.1530
**Smoker**	22 (42.3%)	10 (34.5%)	12 (52.2%)	0.1997
**Coronary artery disease**	21 (40.4%)	13 (44.8%)	8 (34.8%)	0.5729
**Diabetes**	6 (11.54%)	2 (6.9%)	4 (17.4%)	0.3870
**Hypertension**	47 (90.4%)	27 (93.1%)	20 (87.0%)	0.6443
**Chronic kidney disease**	7 (13.5%)	2 (6.9%)	5 (21.7%)	0.2192
**Procedure characteristics**				
**Operation time, min**	401.3 ± 99.0	403.4 ± 96.4	398.7 ± 103.9	0.8680
**Total ventilation time, min**	980 (IQA 570–1980)	1212 (IQA 630–2372)	885 (IQA 485–1590)	0.2351
**In-hospital stay, days**	21 (IQA 11–32)	26 (IQA 18–37)	13.5 (IQA 9–23)	0.2518
**ICU stay, days**	3 (IQA 1–7)	5 IQA 1.5–7)	2 (IQA 1–5)	0.1060
**Baseline MIF, ng/mL**	1.40 (IQA 0.63–2.63) (Min: 0.09, Max: 12.75)	1.09 (IQA 0.51–2.12) (Min: 0.09; Max: 10.45)	1.93 (IQA 1.0–2.65) (Min: 0.24; Max: 12.75)	0.1439
**Baseline serum creatinine, mg/dL**	0.99 (IQA: 0.86–1.20)	0.92 (IQA: 0.85–1.05)	1.14 (IQA: 0.91–1.28)	0.0389 *
**Morbidity and mortality**				
**AKI**	14 (26.9%)	8 (27.6%)	6 (26.1%)	0.2449
**Need for temporary dialysis**	11 (21.2%)	5 (17.2%)	6 (26.1%)	0.5066
**Permanent need for dialysis**	3 (5.7%)	2 (6.8%)	1 (4.3%)	1
**SCI**	3 (5.7%)	2 (6.8%)	1 (4.3%)	1
**Myocardial infarction**	0	0	0	-
**Pneumonia**	10 (19.2%)	7 (24.1%)	3 (13.0%)	0.48
**Tracheotomy**	10 (19.2%)	7 (24.1%)	3 (13.0%)	0.4815
**Sepsis**	6 (11.5%)	4 (13.7%)	2 (8.6%)	0.68
**Surgical revisions**	6 (11.5%)	4 (13.7%)	2 (8.6%)	0.68
**90-Day mortality**	5 (10.42%)	4 (13.8%)	1 (4.4%)	0.3686

If data was missing, the included sample size is reported for the corresponding parameter. Data are reported as absolute numbers and percentages, as mean ± SD and range, or as median with Q1 and Q3, if data were skewed. * *P* < 0.05. ^#^ The *P*-value corresponds to comparison of the expectation value, respectively probability, of the presented row parameter between the surgery methods.

**Table 2:** Univariate analysis of continuous and categorical variables correlating with MIF.

Univariate Analysis of log(MIF) over Time					
	Den DF	*F* Value	*P*-Value	Slope Estimator	SD (Estimator)
Continuous variable					
Age	47.7	0.03	0.8604	−0.00239	0.01351
BMI	47.8	0.34	0.5605	−0.02082	0.03551
Baseline MIF, logarithmized	42.2	11.44	0.0016 *	0.4456	0.1317
Categorical variable					
Gender (male)	48	0.07	0.7942	−0.0857	0.3267
Endovascular repair	48.2	0.01	0.9172	−0.02937	0.2809
Continuous variable (repeated measurements)					
Serum creatinine	171	3.11	0.0796	0.2094	0.1187
Apache II	137	12.92	0.0005 *	0.04273	0.01189

Univariate analysis of the longitudinal model (linear mixed model) with the target variable logarithmized MIF-level 60 min after clamping/contrast solution application (time point 4) using a Kenward Rogers adjustment for small sample size (DF: degree of freedom, NUM: numerator, DEN: denominator; Maximum Likelihood slope estimates with standard deviation (SD); APACHE II: Acute Physiology And Chronic Health Evaluation).* *P* < 0.05.

**Table 3:** Multivariate analysis of different factors with influence on the MIF-levels.

	Den DF	*F*-Value	*P*
Operation method (endovascular)	41.5	0.92	0.3421
Baseline value (MIF), logarithmized	41.3	12.5	0.001 *
Time point (Reference: time point 4)	217	12.37	<0.0001 *
Different time points during surgery	217	2.65	0.0121 *

(DF: degree of freedom, NUM: numerator, DEN: denominator). Time point 4: 60 min after clamping/contrast solution application.* = significant.

## Supplementary Materials

The following are available online at www.mdpi.com/1422-0067/18/11/2374/s1.

## Abbreviations

AG	Study concept, data collection, informed consent, statistical analysis, interpretation, writing, revision
CS	Statistical analysis, writing, revision
AF	Statistical analysis, writing, revision
TS	Interpretation, revision
LM	Data collection, revision
JK	Revision
GS	Data collection, revision
GM	Study concept, informed consent
MJ	Study concept, informed consent
JG	Statistical analysis, writing, revision
