# Supplementary material for: Macrophage Migration Inhibitory Factor Predicts Outcome in Complex Aortic Surgery
Source: Int J Mol Sci. 2017 Nov 9;18(11):2374. doi: 10.3390/ijms18112374 (PMC5713343; doi:10.3390/ijms18112374)
Supplement: Supplementary file 1 [file ijms-18-02374-s001.pdf]

Time	OP	Median	Mean	SD	Lower 95%CL	Upper 95%CL
1	endo	0.45	0.87	2.94	-0.54	2.29
	open	0.69	0.80	1.76	0.04	1.56
2	endo	0.41	0.35	3.20	-1.15	1.84
	open	2.85	3.98	4.84	1.89	6.07
3	endo	0.61	0.15	3.31	-1.49	1.80
	open	2.34	3.53	4.26	1.73	5.33
4	endo	0.42	0.31	3.24	-1.25	1.87
	open	3.02	4.36	5.57	1.96	6.77
5	endo	0.69	0.13	3.28	-1.45	1.71
	open	2.43	3.83	5.37	1.45	6.21
6	endo	0.14	-0.50	2.74	-1.78	0.79
	open	0.36	1.59	3.53	0.03	3.16
7	endo	-0.32	-0.22	2.02	-1.16	0.73
	open	0.03	1.00	3.88	-0.68	2.67
8	endo	-0.40	0.03	2.26	-1.06	1.12
	open	-0.07	0.93	3.55	-0.64	2.50
9	endo	-0.15	0.43	3.06	-1.04	1.91
	open	-0.00	0.30	2.58	-0.88	1.47
10	endo	0.17	-0.05	1.99	-1.01	0.92
	open	0.21	0.46	2.22	-0.53	1.44
11	endo	-0.37	-0.72	2.10	-1.70	0.27
	open	0.05	-0.37	2.23	-1.33	0.59

Table S1 : Description of MIF level change from baseline (MIF-diff) in the endovascular and open surgery subgroup (SD: standard devation). Time points: Baseline; before, 30 minutes after, and 60 minutes after clamping or contrast-solution application; end of procedure; ICU admission; and at 6, 12, 18, 24, 48, and 72 h after ICU admission.

**time point**	**Num DF**	**Den DF**	**F-statistic**	***p***
4	1	69.4	3.44	0.0677
**5**	**1**	**70.2**	**5.31**	**0.0242**
6	1	69.0	1.93	0.1688
7	1	68.3	0.00	0.9864
8	1	69.9	0.03	0.8710
9	1	70.7	0.04	0.8383
10	1	69.9	0.52	0.4752
11	1	68.3	0.21	0.6511

Table S2: Comparison of the mean MIF-levels in the endovascular and the open surgery group (DF – degree of freedom, NUM: numerator, DEN: denominator)

	Comparison of MIF-levels and AKI after endovascular TAAA repair	Comparison of MIF-levels and AKI after open TAAA repair
	n = 15 (no AKI) vs. n = 5 (AKI)	n = 19 (no AKI) vs n= 8 (AKI)
**Time point**	**p (t-approx.)**	**p (t-approx.)**
0	0.7445	0.7853
4	0.2366	0.7328
5	0.7141	0.9781
7	0.2660	0.2047
9	0.3813	**0.0476**
10	0.9675	0.2586

Table S3: Separate comparison of elevated MIF-levels and AKI after endovascular and open TAAA repair.). Time points: Baseline; and 60 minutes after clamping or contrast-solution application; end of procedure; and at 6, 18, 24, h after ICU admission.

Multivariable analysis of log(MIF) over time					
**Covariables**	**Slope Estimate**	**SD(Estimate)**	**DF resp. Num DF/Den DF**	**t- resp. F-value**	**p-value**
**Intercept**	1.1269	0.213	67.3	5.29	<.0001
**Log (MIF) at Baseline**	0.4737	0.134	41.3	3.54	0.0010
**Surgery Method** (Endo)	-0.6022	0.3245	69.4	-1.86	0.0677
**Time Point (overall)**			7/217	12.37	<0.0001
Admission to ICU	-0.04105	0.1677	91	-0.24	0.8072
6h after surgery	-0.6193	0.1673	93.4	-3.7	0.0004
12h after surgery	-0.9781	0.1659	94.8	-5.9	<.0001
18h after surgery	-0.8726	0.1675	103	-5.21	<.0001
24h after surgery	-1.0443	0.1682	117	-6.21	<.0001
48h after surgery	-0.8712	0.1623	142	-5.37	<.0001
72h after surgery	-1.0127	0.1419	115	-7.14	<.0001
**Surgery method by Time Point interaction** (Endo*TP)			7/217	2.65	0.0121
Endo*Admission to ICU	-0.1478	0.2557	146	-0.58	0.5642
Endo*6h after surgery	0.1519	0.2537	149	0.6	0.5503
Endo*12h after surgery	0.5967	0.2526	155	2.36	0.0194
Endo*18h after surgery	0.5493	0.2546	174	2.16	0.0323
Endo*24h after surgery	0.669	0.2529	209	2.65	0.0088
Endo*48h after surgery	0.3689	0.2425	261	1.52	0.1293
Endo*72h after surgery	0.4555	0.2075	223	2.19	0.0292

Table S 4: *Target variable: Logarithmized MIF from Reperfusion (time point 4).Reference category of time measurement, open surgery reference category of surgery method). (DF – degree of freedom, NUM: numerator, DEN: denominator, Maximum Likelihood slope estimates with standard deviation (SD); *: p<0.05.*

Open surgery

Open surgical repair was performed under general anesthesia. Cerebrospinal fluid (CSF) pressure was assessed using intrathecal catheter and maintained during the procedure and in the intensive care unit for 72 hours. CSF was allowed to drain spontaneously if pressure increased above 10 mmHg. Spinal cord function was monitored as described before (1). After thoracolaparomtomy, exposure of the aorta and heparinisation (0.5 mg/kg; activated clotting time [ACT] approximately 600 seconds), extracorporeal circulation with a femoro-femoral cannulation was established for each type of TAAA repair. While clamping the viscerorenal segment, the visceral arteries were perfused with selective catheters providing > 500 ml/min per catheter.. The renal arteries were perfused with 4°C tempered cardioplegia solution (Custodiol, Dr. Franz Köhler Chemie, Austria). This maneuver had been described to protect the kidneys from ischemic organ damage (2). Further details about the surgical procedure have been reported by Jacobs et al (3, 4)

Endovascular surgery

Endovascular repair was performed via femoral and axillary access using fenestrated and branched components to include the viscera-renal vessels in the reconstruction under general anesthesia. Further details about the endovascular procedure have been reported before (5). A reduced dose of iodinated contrast solution was used (1/4 of the standard dose) for the selective renal artery angiography to reduce the risk of contrast-solution induced AKI (6).

1. Jacobs MJ, Elenbaas TW, Schurink GW, Mess WH, Mochtar B. Assessment of spinal cord integrity during thoracoabdominal aortic aneurysm repair. Ann Thorac Surg. 2002;74(5):S1864-6; discussion S92-8.
2. Tshomba Y, Baccellieri D, Mascia D, Kahlberg A, Rinaldi E, Melissano G, et al. Open treatment of extent IV thoracoabdominal aortic aneurysms. J Cardiovasc Surg (Torino). 2015;56(5):687-97.
3. Kalder J, Kokozidou M, Keschenau P, Tamm M, Greiner A, Koeppel TA, et al. Selective renal blood perfusion induces renal tubules injury in a porcine model. J Vasc Surg. 2016;63(3):778-87.
4. Jacobs MJ, Mommertz G, Koeppel TA, Langer S, Nijenhuis RJ, Mess WH, et al. Surgical repair of thoracoabdominal aortic aneurysms. J Cardiovasc Surg (Torino). 2007;48(1):49-58.
5. O'Brien N, Sobocinski J, d'Elia P, Guillou M, Maioli F, Azzaoui R, et al. Fenestrated endovascular repair of type IV thoracoabdominal aneurysms: device design and implantation technique. Perspect Vasc Surg Endovasc Ther. 2011;23(3):173-7.
6. Canyigit M, Cetin L, Uguz E, Algin O, Kucuker A, Arslan H, et al. Reduction of iodinated contrast load with the renal artery catheterization technique during endovascular aortic repair. Diagn Interv Radiol. 2013;19(3):244-50.
